# Notes and Comments

**Published:** 1893-06

**Authors:** 


### Notes and Comments.[1]

One of the best measures of the progress of man is the degree of his ability to stand alone, in thought and action, undisturbed, though he may often profit by the adverse opinions and judgments of others. "When the people are a herd they are easily swayed and ruled by one man; when they are individualized, the dominion of one is not possible." Let us hold, then, and teach that better than great riches and possessions is a brave heart, an enlightened and well-balanced mind, an appreciative, hopeful, and helpful soul. Let our labors be a sort of religion, urging us to elevation, seriousness, and chastity of thought and actions.

To quote from Charles Kingsley: "Men can be as original now as ever, if they had but the courage, even the insight. Heroic souls in old times had no more opportunities than we have; but they used them. There were daring deeds to be done then,—are there none now? Sacrifices to be made,—are there none now? Wrongs to be redressed,—are there none now?"

[1] The assistant editor solicits contributions for this department,—new methods, new remedies and formulas, or any short practical note which may prove of value to the practitioner or student. Address 1506 Arch Street, Philadelphia.

Implantation.—While discussing a paper by Dr. Younger upon "Some of the Latest Phases in Implantation," Dr. George S. Allan made the following pointed remarks:

"No biologist of the present time questions the law, *omne vivum ex ovo*, nor would he hesitate longer to give his support to the assertion that no dead matter, though once living matter, can again become living matter except through a process of digestion and assimilation through a living body. Such a thought as that Dr. Younger suggests—that a mass of dead matter can, as you might say, without breaking bulk or altering shape, become again living matter—would only excite laughter or derision in the mind of the true student of vital powers and conditions and their origin.

"I wonder that Dr. Younger did not submit his paper to some one having correct knowledge of the fundamental biological laws he would overturn in his anxiety to sustain a false position,—a position, by the bye, not at all necessary to assume in order to support his well-merited claims in bringing to new life the possibilities of the system of implantation."

---

The Clumsy Dentist.—Among other good things, friend Welch, in the editorial pages of the *Items of Interest*, says,—

"How clumsily the blundering dentist stumbles along in his difficult work, seeking unmerited success. He complains of his awkward instruments and of his fractious materials,—of the unmanageableness of everything but himself. They do not obey him because he does not know how to command them. He is the servant of circumstances and the master of nothing. How he fumes and frets at his hard lot, when he ought to see that *he is only spoiling a good ploughman to make a poor dentist*."

---

"The Cobalt of Herbst."—After making numerous experiments with the material, Dr. E. C. Kirk says, "It is unquestionably a fact that the cobalt of the Herbst method for treating pulps is not what is now known to chemists as cobalt, but pure metallic arsenic, and nothing else. Whether the cocaine hydrochlorate, of which eight per cent. is added, has any function apart from its usual anæsthetic property, is not clear, but it may easily be that it is to a certain extent decomposed, giving up its chlorine to a portion of the arsenic, and so forming an arsenic chloride which, being soluble, would be more readily absorbed by the pulp. It is also possible that the free acid which most of the samples of cocaine hydrochlorate are said to contain may also help to increase the solubility of the metallic arsenic. Be that as it may, it is well for us to recognize, before adopting this method, that whatever of value it may have is due to the effect of arsenic, and not in the remotest degree to cobalt.

"As to the effects of arsenic upon an exposed pulp the dental profession are pretty familiar. How far they may be modified as to the final result by using metallic arsenic instead of its oxide remains to be seen, but it does not seem difficult to guess."

